# From Poly(glycerol itaconate) Gels to Novel Nonwoven Materials for Biomedical Applications

**DOI:** 10.3390/gels9100788

**Published:** 2023-09-29

**Authors:** Magdalena Miętus, Krzysztof Kolankowski, Tomasz Gołofit, Piotr Denis, Aleksandra Bandzerewicz, Maciej Spychalski, Marcin Mąkosa-Szczygieł, Maciej Pilarek, Kamil Wierzchowski, Agnieszka Gadomska-Gajadhur

**Affiliations:** 1 Faculty of Chemistry, Warsaw University of Technology, Noakowskiego 3 Street, 00-664 Warsaw, Poland; magdalena.mietus.dokt@pw.edu.pl (M.M.); krzysztof.kolankowski.dokt@pw.edu.pl (K.K.); tomasz.golofit@pw.edu.pl (T.G.); aleksandra.bandzerewicz.dokt@pw.edu.pl (A.B.); 2 Laboratory of Polymers and Biomaterials, Institute of Fundamental Technological Research, Polish Academy of Sciences, Pawińskiego 5B Street, 02-106 Warsaw, Poland; pdenis@ippt.pan.pl; 3 Faculty of Materials Science and Engineering, Warsaw University of Technology, Wołoska 141 Street, 02-507 Warsaw, Poland; maciej.spychalski@pw.edu.pl; 4 Department of Chemistry, Faculty of Natural Sciences, Norwegian University of Science and Technology, 7034 Trondheim, Norway; marcin.k.makosa-szczygiel@stud.ntnu.no; 5 Faculty of Chemical and Process Engineering, Warsaw University of Technology, Waryńskiego 1 Street, 00-645 Warsaw, Poland; maciej.pilarek@pw.edu.pl (M.P.); kamil.wierzchowski@pw.edu.pl (K.W.)

**Keywords:** poly(glycerol itaconate), polylactide, electrospinning, tissue engineering, hydrogels

## Abstract

Electrospinning is a process that has attracted significant interest in recent years. It provides the opportunity to produce nanofibers that mimic the extracellular matrix. As a result, it is possible to use the nonwovens as scaffolds characterized by high cellular adhesion. This work focused on the synthesis of poly(glycerol itaconate) (PGItc) and preparation of nonwovens based on PGItc gels and polylactide. PGItc gels were synthesized by a reaction between itaconic anhydride and glycerol. The use of a mixture of PGItc and PLA allowed us to obtain a material with different properties than with stand-alone polymers. In this study, we present the influence of the chosen ratios of polymers and the OH/COOH ratio in the synthesized PGItc on the properties of the obtained materials. The addition of PGItc results in hydrophilization of the nonwovens’ surface without disrupting the high porosity of the fibrous structure. Spectral and thermal analyzes are presented, along with SEM imagining. The preliminary cytotoxicity research showed that nonwovens were non-cytotoxic materials. It also helped to pre-determine the potential application of PGItc + PLA nonwovens as subcutaneous tissue fillers or drug delivery systems.

## 1. Introduction

Electrospinning is a process that dates back to the 1930s [1,2]. Electrospinning allows the preparation of continuous polymer fibers with the use of a high electric field [3]. The process is based on the deformation of a droplet of the spun material to form a Taylor cone and results in the formation of fibers [3,4,5]. The obtained fibers can be as thin as a few nanometers in diameter [6,7,8]. The most significant components of an electrospinning apparatus are as follows: capillary tube with small needle/pipette, high-voltage supplier and collector (fix collector or rotary collector) [9]. The most significant qualities of the electrospinning process and the resulting fibers are (dependent on polymer–solvent configuration) as follows:

- The ability to produce flexible, three-dimensional cellular scaffolds with high porosity. This allows for a large specific surface area [1,3,7,8,10,11,12]. The fibers’ structure increases the capacity of the produced fibers [13]. A high surface-to-volume ratio characterizes fibers [6,10].
- The ability to admix the fibers and substances with antibacterial and anti-inflammatory properties [5,14].
- The potential to promote the growth and differentiation of different cell types [5,15]. The structure of the fibers provides an easy exchange of nutrients and metabolic wastes between the scaffold and the environment [16].
- Capability of implementing the drug on the fibers’ surface or inside the fibers [11,17].
- The ability to maximize the effect of the drug implanted in the fibers. This is a result of the controlled and continuous release of the drug at the intended site [11,18].
- The potential to form fibers in a wide variety of shapes and sizes [6]. The produced fibers are almost identical in their diameter [7,8].
- Mild processing conditions (atmospheric pressure, room temperature) [6].
- Relatively simple procedure for the production of fibers [5,19,20,21].
- The potential to decrease the risk of inflammation at the implantation area (by releasing drugs that are effective against antibiotic-resistant bacteria) [11,22].

The number of articles and patents covering this process is continuously increasing [3,9,23,24]. This is due to the applicability of the process primarily in medicine, tissue engineering (bone tissue engineering, cartilage tissue, skin tissue, the nervous system and the circulatory system) and the military industry [5,9,25,26,27,28]. Electrospun fibers are suitable for use in devices such as sensors, optical sensors and filter membranes, or for manufacturing protective clothing [6,11,26,29]. Tissue engineering is an interdisciplinary field of science that provides the ability to produce materials that act like natural tissues (like ECMs—Extracellular Matrices) [1,30,31,32,33,34,35]. As a result, cell fusion and cell differentiation may be easier, and cell viability higher [11,12]. In the presented article, the chosen electrospinning method was the one that resulted in randomly oriented nonwovens. In the future, it will be interesting to carry out the electrospinning process with radial alignment. According to literature reports, such an arrangement should allow efficient implementation of cells. It should also improve their proliferation [36].

The electrospinning process uses natural and synthetic polymers to produce high-quality polymer fibers. These include gelatin (Gel), chitosan, collagen, poly(ε-caprolactone) (PCL), polylactide (PLA) and poly(lactide-co-glycolide) copolymer (PLGA) [6,16,26,37]. An example of the use of electrospinning process in medicine is a composite of polylactide, silk and gelatin or a PCL-based material for producing tubular blood vessel substitutes [28,38]. A mixture of PCL and PLGA copolymer has the potential to regenerate cartilage tissue [1,39]. The poly(glycerol sebacate) (PGS) and PCL blend fibers allow the regeneration of cardiac tissues [1,40]. There is a high level of interest in the materials for skin tissue regeneration [1,41]. One example is gelatin and starch-based nanofibers [41]. They promote the healing of second-degree burn wounds and reduce the body’s inflammatory response [41].

In the literature, there is a shortage of articles on the production of nonwovens in which poly(glycerol itaconate) is one of the components. Nevertheless, some articles cover the production of other polyester fibers. In 2019, a research group directed by T. Ahmadi conducted studies on the preparation and characterization of nano-fibers from polycaprolactone fumarate (PCLF) and Gel alongside a small addition of fluorapatite (FA) [42]. FA was doped with silicon and magnesium (Si-Mg-FA) [42]. The material was produced to investigate its potential use in bone tissue regeneration [42]. A 5% addition of Si-Mg-FA nanoparticles to PCLF-Gel improved the mechanical properties of the obtained nanocomposites [42]. An important feature was the non-cytotoxicity of the produced fibers [42]. Another polymer composite prepared by electrospinning was a biocompatible poly(glycolic acid-co-propylene fumarate) [43]. It contained graphene oxide (GO), which has antibacterial properties [43]. PPF made it possible to obtain a material with mechanical properties similar to trabecular bone [43]. PPF was also used as one of the poly(propylene fumarate-co-propylene maleate) copolymer (PPFcPM) components [44]. The fibers produced from this material are expected to be examined for their use as scaffolds for bone tissue engineering applications [44]. In 2016, I. Romano et al. succeeded in producing nanofibers based on poly(octyl cyanoacrylate) (POCA) and poly(propylene fumarate) for medical purposes [45]. The achieved material is supposed to help regenerate skin tissue damaged by burns [45]. The presence of PPF resulted in the anti-inflammatory properties of the material and restoration of the original skin conditions [45]. Glycerol, the hydrophilic alcohol, was one of the components of an electrospun poly(glycerol dodecanedioate-co-fumarate) copolymer (PGDF) [46]. It was synthesized in the reaction of glycerol, dodecanedioic acid and fumaric acid [46]. The received product was a non-toxic, biocompatible material [46]. Currently, there is on-going research on the use of this material in tissue engineering of the neural system [46].

In this work, we have prepared nonwoven fabrics from two synthetic polymers: poly(glycerol itaconate) (PGItc) and PLA, in different proportions of the used polymers [6]. A mixture of these two polymers was used to reduce the hydrophobicity of PLA—an increase in cell adhesion was expected to occur. The purpose of this article was to produce and analyze PGItc + PLA fibers. It should allow us to determine the area of their potential application in tissue engineering—as subcutaneous tissue fillers, in drug delivery systems, patches, etc. The double bond in PGItc allows the mechanical properties of the resulting nonwovens to be modified as needed.

The use of a mixture of polymers enables the production of better scaffolds for cell implementation [26]. For instance, improvements in the thermal stability, mechanical strength and barrier properties of these materials have been shown [6]. Furthermore, producing self-contained fibers from PGItc is challenging, as the resulting PGItc had a low molecular weight [42].

**Figure 1:**
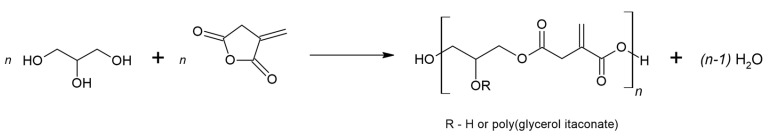
Synthesis of PGItc from glycerol and itaconic anhydride.

Poly(glycerol itaconate) is an unsaturated polyester relatively poorly characterized in the literature. It can be synthesized by a polycondensation reaction between itaconic acid/anhydride and glycerol (Figure 1) [47].

PGItc is a non-toxic macromolecule because of the non-toxicity of the substrates used to produce it [48,49]. Another important advantage of PGItc is the ability to synthesize it without the use of a catalyst or solvent [47]. Both itaconic acid and glycerol are used in the pharmaceutical, cosmetic and medical industries [48,49,50,51]. PGItc is remarkable due to the presence of a C=C double bond in the side chain, which enables the post-polymerization reactions to take place. As a result, it is possible to develop products with a wide range of properties [52,53,54].

Itaconic compounds are frequently used to obtain hydrogel materials [55,56]. For instance, hydrogels based on Laptonite^®^ and high-molecular-weight poly(itaconic acid) were obtained [56]. They will potentially be used in drug-delivery systems and artificial muscles [56]. Meanwhile, in the article [55], various methods of obtaining hydrogels based on poly(ethylene glycol) and poly(itaconic acid) were studied for the ability to change shape depending on the pH of the environment. In the future, these hydrogels will be investigated for their use in oral drug delivery [55].

The second component of the produced fibers, PLA, is a biodegradable polymer with good mechanical and biological properties [38,57]. PLA degrades to carbon dioxide and water in less than 90 days under in vitro conditions [58]. Under in vivo conditions, PLA degradation time can extend to several months [59]. This property allows the development of orthopedic components with a sufficiently long service life [59].

## 2. Results

### 2.1. PGItc Gel Characterization

**Figure 2:**
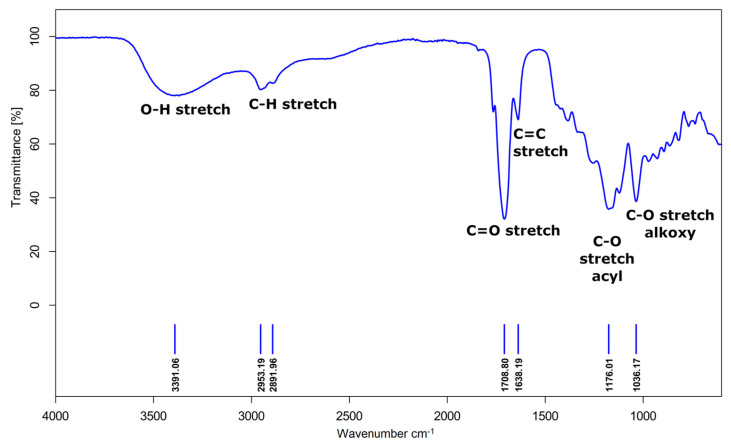
FTIR spectra of poly(glycerol itaconate) gel (T = 140 °C, t = 4 h, functional group ratio OH/COOH—1.5).

**Figure 3:**
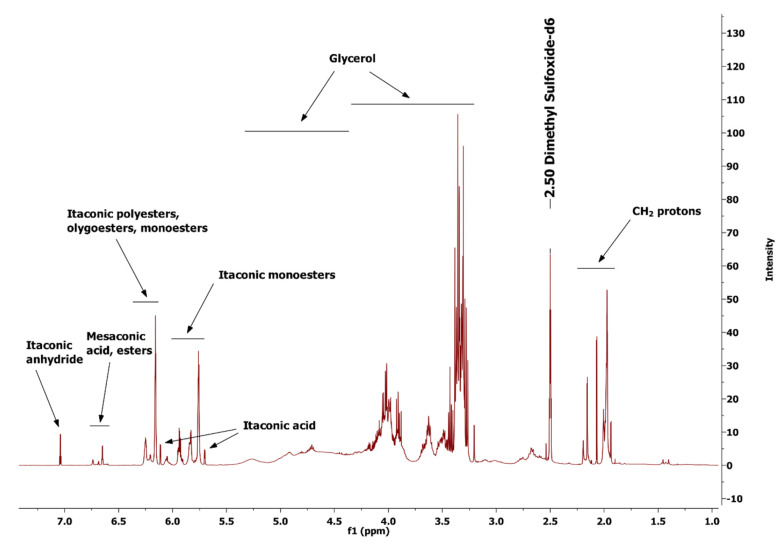
^1^H NMR spectra of poly(glycerol itaconate) gel (T = 140 °C, t = 4 h, functional group ratio OH/COOH–1.5).

The chemical structure of poly(glycerol itaconate) gels (Figure S1) was characterized by the analysis of FTIR (Figure 2) and NMR spectra (Figure 3).

The presence of polyester is demonstrated by:

- The band representing the stretching vibrations of the C=O carbonyl group (1709 cm^−1^);
- The stretching vibrations band of the C-O acyl group (1176 cm^−1^);
- The stretching vibrations band of the C-O alkoxy group (1036 cm^−1^).

The FTIR spectrum also shows hydroxyl group vibrations (3391 cm^−1^), vibrations of C-H bonds in the main aliphatic chain (2953 cm^−1^ and 2892 cm^−1^) and the vibrations of the unsaturated C=C bond (1638 cm^−1^).

Figure 3 presents the ^1^H NMR spectrum of the obtained PGItc gel with the interpretation of the assigned proton signals.

Figure 3 shows signals from unreacted substrates and the obtained product. Itaconic anhydride protons are present in the 7.05–7.03 ppm range. Numerous signals from the desired product, PGItc gel, are visible on the spectrum. In addition, there are visible signals from one of the isomers of itaconic acid, mesaconic acid, and esters formed in the reaction of mesaconic acid with glycerol. The signals in the ranges 5.40–4.40 ppm and 4.30–3.25 ppm are the protons of glycerol. The signals in the 4.30–3.25 ppm range are the protons from the unreacted glycerol. The signals in the 2.25–1.95 ppm range are from the CH_2_ protons.

### 2.2. SEM Images Analysis

**Figure 4:**
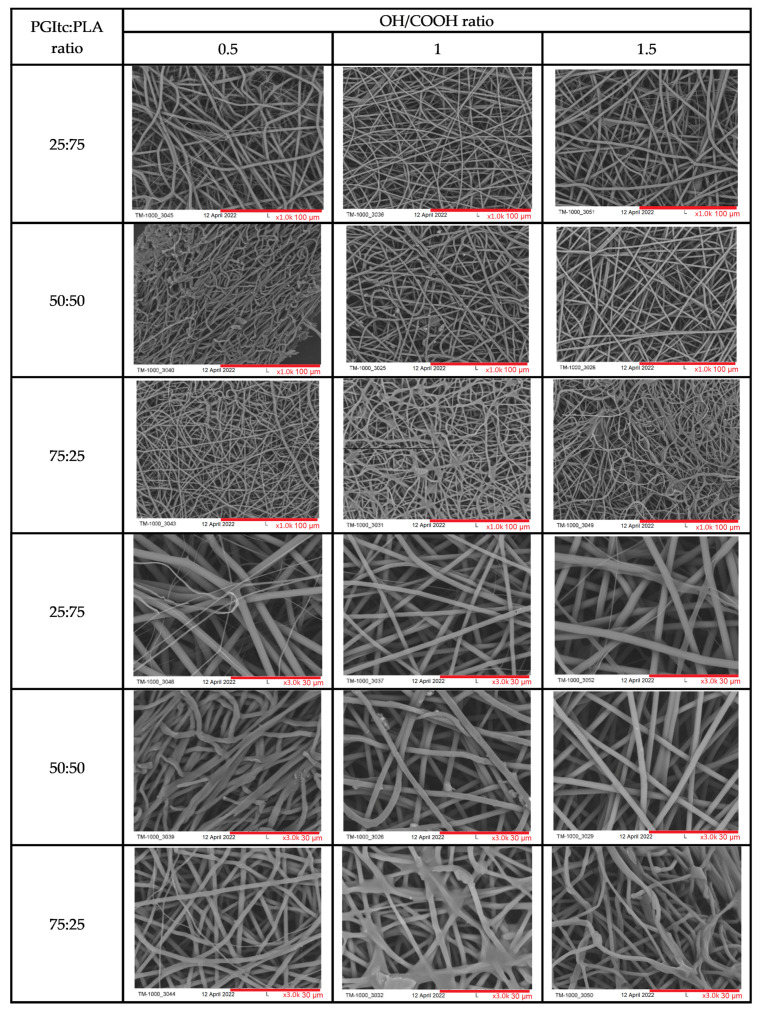
SEM images of produced nonwovens (red lines refer to the scale).

Regardless of the used OH/COOH ratio in the PGItc gel synthesis procedure, the fibers are randomly oriented (Figure 4). The parameters of the conducted electrospinning process (voltage applied) and the concentration of the individual polymers influenced the diameter of the prepared fibers and their surface structure. The waviness of the fibers changed with a change in the ratio of the used polymers. The waviest fibers were produced when the ratio of reactants was 75:25 (PGItc:PLA). The pores between the fibers are small in size, which could be a result of the low molecular weight of the PGItc gels. When the polymer ratio was 50:50 and the functional group ratio was 1, beads were visible on the resulting fibers, representing unreacted itaconic anhydride crystals. The remaining fibers had a smooth and regular surface topography. The beads were not visible.

Table 1 summarizes the average values of the diameters of the received fibers. The fiber diameter was the smallest when there was a great excess of PGItc gel (range 1.26–1.74 μm). The smallest fiber diameter occurred at a ratio of hydroxyl groups to carboxyl groups of 1.5 (1.26 ± 0.14 μm). Higher hydrophobic PLA content resulted in fibers with an increased diameter. The reason for this is the higher viscosity of the material (higher viscosity resistance).

The fiber diameter dispersion remained small for particular PGItc:PLA ratios (exception: PGItc:PLA 25:75, OH/COOH = 1). The concentration of PGItc in the solution significantly influenced the diameter of the produced fibers. In most cases, an increase in the concentration of hydroxyl groups compared to carboxyl groups reduced the diameter of the obtained fibers.

### 2.3. Water Contact Angle Analysis

**Figure 5:**
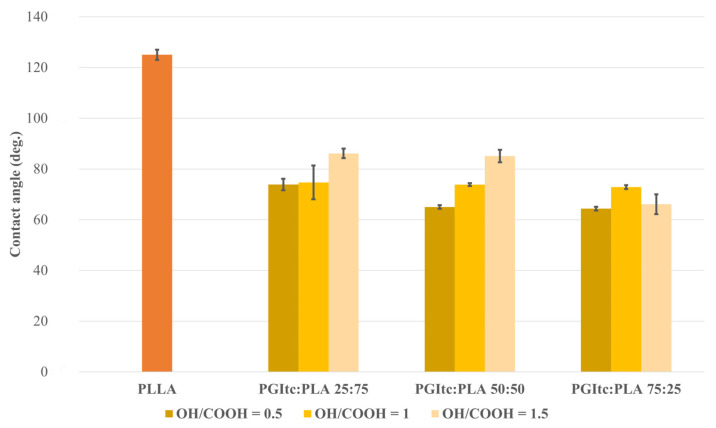
Water contact angle value for prepared nonwovens. The wetting angle for PLLA was taken from [60].

Water contact angle measurements were conducted (Figure 5), as this is an important property of biomaterials. It can influence cell attachment, proliferation, migration and cell viability. PLA is a material with hydrophobic properties. Due to the presence of glycerol-derived hydroxyl groups, PGItc is a hydrophilic material. All the obtained nonwovens had water contact angle values θ < 90°. In other words, the nonwovens were characterized as hydrophilic. The achieved surfaces tended to wet out and form a thin film. The low water contact angle values indicate the high porosity of the obtained nonwovens. This is highly relevant in terms of the potential application of fibers as medical dressings. The higher content of PGItc gel in relation to polylactide leads to a lower water contact angle. This indicates the greater hydrophilic properties of the fibers and leads to the conclusion that such fibers could be successfully used for cell culture. Interestingly, for PGItc:PLA ratios of 25:75 and 50:50, the higher the OH/COOH ratio, the greater the contact angle. Glycerol is miscible with water, so the higher the glycerol content, the smaller contact angle value should be. The received results may be caused by the formation of short clusters of PGItc gel oligomers. High chain entanglement may contribute to the obscuration of the hydroxyl groups of glycerol.

### 2.4. Cytotoxicity Analysis

**Figure 6:**
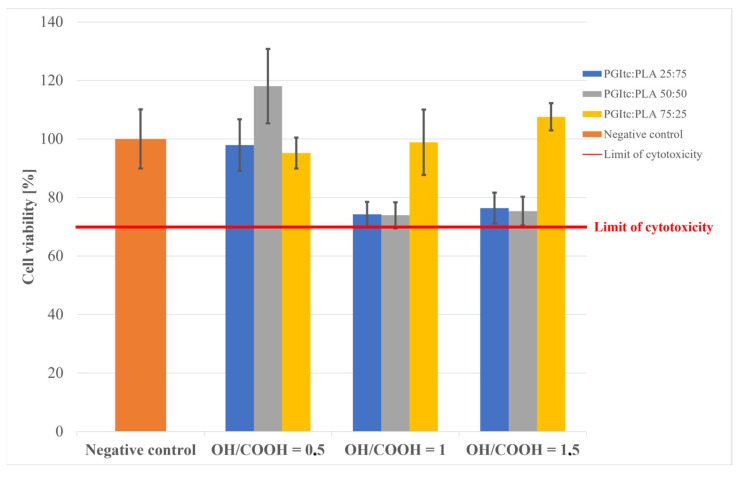
The results of the nonwoven cytotoxicity tests.

For all tested materials, cell viability was above the cytotoxicity limit (70% of negative control, Figure 6). The OH/COOH molar ratio in PGItc strongly affected the results. In the case of the excess of COOH groups, the material was probably degrading faster, resulting in culture media acidification. Still, the pH drop was not significant enough to be considered cytotoxic. The viability rates generally increased along with the amount of PGItc gel in the nonwovens. 

### 2.5. Optical Profilometer Surface Imaging

**Figure 7:**
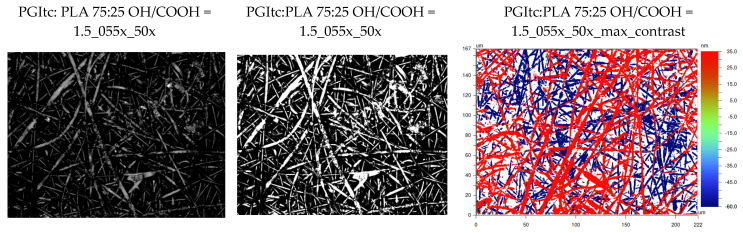
Profilometer analysis of produced nonwovens.

The profilometric analysis confirmed the results of the SEM analysis of the produced fibers (167 × 222 μm) (Figure 7). The material’s surface consists of densely packed, randomly arranged fibers (R_a_ = 3.609 μm). In the obtained profilometric images, two layers of fibers can be observed. The layers are 95.0 nm distant from each other.

### 2.6. Crosslinking and Leaching of the Nonwovens

**Figure 8:**
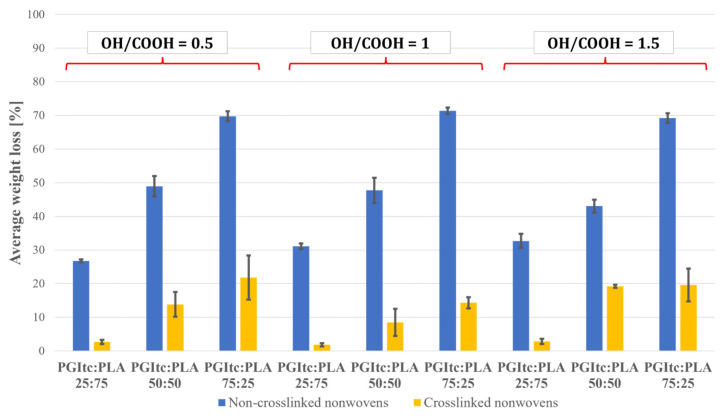
The results of the non-crosslinked and crosslinked nonwovens leaching tests.

The effectiveness of the thermal crosslinking of the nonwovens obtained was tested (Figure 8). The crosslinking efficiency was determined by the weight loss of the crosslinked nonwovens. The lower the weight loss of the nonwovens, the higher the crosslinking efficiency of the nonwovens. The results for crosslinked nonwovens were compared with those obtained for non-crosslinked nonwovens.

Based on the study, a significant excess of PGItc gel (PGItc:PLA 75:25) resulted in the highest weight loss for non-crosslinked and crosslinked nonwovens. The lowest weight loss of non-crosslinked and crosslinked nonwovens occurred when a small amount of PGItc gel (PGItc:PLA 25:75) was used. The weight loss of non-crosslinked and crosslinked nonwovens slightly depended on the ratio of hydroxyl groups to carboxyl groups. The average weight loss values received show similar values for the PGItc:PLA ratios analyzed. Comparing the mass loss of non-crosslinked and crosslinked nonwovens, it can be observed that the mass loss of crosslinked nonwovens is significantly lower than that of non-crosslinked nonwovens. This makes it possible to conclude that the crosslinking process occurred efficiently, and only a small amount of non-crosslinked PGItc was washed out.

### 2.7. DSC Analysis

Several effects can be observed for non-crosslinked and crosslinked nonwovens. Each nonwoven is characterized by the presence of glass transition (T_g_), cold crystallization during heating (T_cc_), cold crystallization during cooling (T_ccc_) of the nonwoven and polymer melting (T_m_). For some of the non-crosslinked nonwovens, two melting temperatures T_m1_ (shoulder-melting) are additionally visible during the first heating. These temperatures are different by a few degrees Celsius. The temperature transformation values for pure PLA were taken from the article of Dai X. et al. (T_g1_ = 50.87 °C, T_cc1_ = 67.57 °C, T_m1_ = 180.80 °C) [61]. Based on the melting enthalpy values of the tested nonwovens and pure PLA (ΔH_m_° = 93 J/g), the degree of crystallinity (X_c_) was determined for each nonwoven [62]. A comparison of the thermal transformations of the nonwovens and pure PLA is presented below.

**Figure 9:**
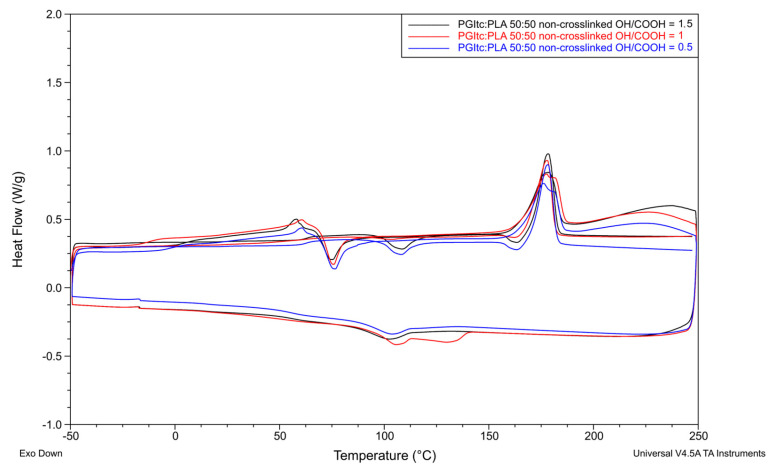
DSC analysis for non-crosslinked nonwovens (PGItc:PLA 50:50).

**Figure 10:**
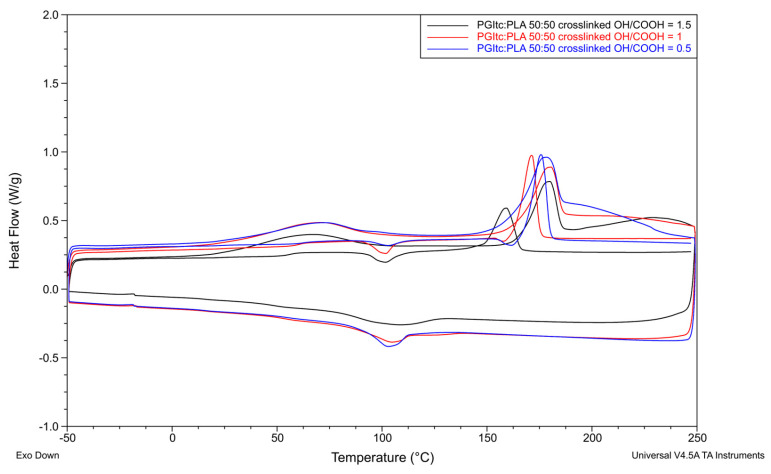
DSC analysis for crosslinked nonwovens (PGItc:PLA 50:50).

A comparison of the thermal transformations of uncrosslinked (Figure 9) and crosslinked (Figure 10) nonwovens for a PGItc:PLA ratio of 50:50, for different OH/COOH ratios, is shown below.

Non-crosslinked nonwovens showed an inflexion from −12.1 °C to 4.6 °C during the first heating. This corresponds to the glass transition temperature T_g1_ and represents the glass transition temperature of PGItc. After that, a weak endothermic inflexion corresponding to the PLA’s glass transition (T_g1′_) was seen. During the second heating of non-crosslinked nonwovens, the observable glass transition temperature (T_g2_) was higher (range of 61.8 °C to 63.5 °C). The glass transition temperature obtained for PLA in the PGItc + PLA composition was higher than for pure PLA. The reason for such a change is the presence of PGItc, which makes it challenging to access PLA chains. The change in content of PGItc gel significantly affected the mobility potential of PLA chains. As the temperature increased, the possibility of polymer mobility rose. Cold crystallization occurred, meaning that the polymer chains underwent conformational changes and became more flexible. For nearly all non-crosslinked nonwovens, an additional exothermic peak—the crystallization peak (T_c_)—occurred before the melting temperature T_m2_ was reached. This is due to the incomplete crystallization of the PLA used. The OH/COOH functional group ratio did not significantly affect the T_cc1_ and T_cc2_ values. The slight differences in the case of the melting point values T_m1_ and T_m2_ of uncrosslinked nonwovens allow us to state that the crystalline structure of the nonwovens is similar. In the case of nonwovens characterized by the presence of two melting temperatures T_m1_, a lower temperature corresponds to a less perfect PLA crystalline structure. The higher temperature T_m1_ corresponds to a more perfect PLA crystalline structure. The OH/COOH ratio has a small effect on the values of these temperatures and the obtained values of the degree of crystallinity of non-crosslinked nonwovens at the same PGItc:PLA ratio.

The used OH/COOH ratio affected the obtained values of the degree of crystallinity of the tested non-crosslinked nonwovens using different PGItc:PLA ratios (Tables S4 and S5). A higher proportion of poly(glycerol itaconate) in the tested nonwovens influenced a decrease in X_c_ values. This is a logical result due to the increasing proportion of amorphous polymer in the tested nonwovens due to the difficulty in organizing the chains of PLA, which is surrounded by a large number of PGItc chains. An interesting feature is the peak corresponding to T_m2_. It is not a double peak, as in the case of the T_m1_ peak. This indicates the occurrence of a reaction between PGItc and PLA chains at an elevated temperature during the first heating.

The differences in glass transition temperature values during the first (T_g1_) and second (T_g2_) heating are not as significant as in the case of the non-crosslinked nonwovens. For this reason, the measured glass transition temperature corresponds to the glass transition temperature of pure polylactide. For an equal PGItc:PLA ratio, the OH/COOH ratio has a stronger effect on T_m1_ and T_m2_ than for uncrosslinked samples. As the proportion of hydroxyl groups increased, the melting points of T_m1_ and T_m2_ decreased. This is due to the formation of many short PGItc chains. In most cases, the crystallinity degree values obtained for crosslinked nonwovens were lower than for non-crosslinked nonwovens. 

For both non-crosslinked and crosslinked nonwovens, there was a difference in the values of enthalpy of cold crystallization (ΔH_cc2_) and enthalpy of melting (ΔH_m2_) during the second heating cycle. The reason for this is that PLA crystals can be reorganized. They undergo continuous melting and recrystallization during heating. No cold crystallization temperature during cooling was observed for one non-crosslinked (PGItc:PLA 75:25, OH/COOH = 1) and two crosslinked samples (PGItc:PLA 75:25, OH/COOH = 1.5 and PGItc:PLA 25:75, OH/COOH = 1).

The DSC analysis provided important information about the effect of crosslinking of the nonwovens on the temperature characteristics. Changes in the values of the glass transition temperatures during the first heating of non-crosslinked and crosslinked nonwoven samples can be seen. The higher the glass transition temperature tested, the higher the degree of crosslinking of the nonwovens. This allows us to conclude that the crosslinking process of the nonwovens took place with high efficiency. The obtained values of characteristic temperatures and enthalpies make it possible to conclude that thermal crosslinking changes the thermal properties of the prepared nonwovens.

## 3. Discussion

The diameter of the obtained fibers is affected by the parameters of the electrospinning process—the molecular weight of the polymer in the solution, the concentration of the used solution or the applied voltage [38]. The fibers’ diameters were larger than those obtained from other glycerol polyesters with PLA [61,63]. Compared to the nonwovens from poly(glycerol succinate) (PGSu) and PLLA, the diameters of the PGItc + PLA fibers decreased more when increasing the proportion of PGItc gel (approx. 1–1.5 μm) [61]. The used concentration of the solution and the applied voltage were different in both cases. This could be the reason for the different diameters of the obtained nonwovens. In addition, the cause for the reduction in fiber diameter could have been the changing viscosity of the spinning solution with the change in PGItc content, as well as structural differences between PGSu and PGItc. The use of citric acid (tricarboxylic acid) as one of the ingredients for the production of fibers—a mixture of poly(glycerol citrate) (PGCit) and PLA—contributed to an increase in the diameter of the obtained fibers [64]. The diameter of the fibers increased with an increase in the content of citric acid [64]. 

Using a polymer similar in structure to PGItc for producing fibers contributed to a decrease in differences in the obtained fiber diameters [46]. Dai X. et al. discussed [46] fibers with fumaric acid (FAc). FAc has a multiple bond in its structure, similar to itaconic anhydride [65]. The diameter of the obtained fibers changed depending on the content of poly(glycerol-dodecanedioate-co-fumarate) (PGDF) in the spinning solution [46]. Although the values of the obtained diameters of fibers with PGDF were similar to those obtained for PGItc + PLA (omitting the influence of using different electrospinning parameters), in the case of PGDF fibers, an increase in the content of PGDF in the spinning solution contributed to an increase in fiber diameter rather than a decrease [46].

When comparing the diameter values of the obtained nonwovens, it is important to note their potential application. The nonwovens obtained from PGDF have been studied for their use in neural tissue engineering [46]. For PGSu-PLLA and PGCit-PLA nonwovens, their specific use in tissue engineering has not been determined. Natural ECM for bone regeneration has pores in the range of 200–350 μm [66]. For skin regeneration, pores should have a maximum diameter of 125 μm, and for blood vessel regeneration, pores should have a maximum diameter of 5 μm [66]. The resulting PGItc + PLA fibers had diameters in the range of 1.12–3.45 μm and the pores were very small. Assuming that the pores in such nonwovens would be similar in size to the diameters of the obtained fibers, they could be used as a potential drug delivery system for skin healing. Verreck G. et al. discussed [67] fibers with diameters of 2 μm. They were produced for use in controlled drug delivery in wound healing and in topical applications [67]. PGItc + PLA nonwovens could also be examined for their application as 3D scaffold models for the study of cancer cells. Cavo M. et al. described [68] a review of research on electrospun materials with applications in cancer research was performed. For example, the obtained PCL nonwoven fibers had diameters in the range of 400 nm to 10 μm, allowing for optimal cancer cell colonization [68]. The presented analysis of the diameters of electrospun fibers suggests that PGItc + PLA fibers are expected to be suitable for application in selected medical fields.

The contact angles of the obtained nonwovens were lower than similar nonwovens obtained from poly(glycerol sebacate) (PGS) and PLLA [63]. PGItc + PLA nonwovens were more hydrophilic than PGS+PLLA nonwovens [63]. This effect increased with the increase in poly(glycerol itaconate) content. It results from the presence of hydroxyl groups in the glycerol structure [69]. The obtained PGItc + PLA nonwoven fabrics were also more hydrophilic than the PGCit-PLA nonwovens [64]. Furthermore, the nonwovens produced from PGCit-PLA were hydrophobic, as their wetting angle in the majority of cases was significantly greater than 90° [64].

Cytotoxicity tests conducted on the PGItc + PLA nonwovens led to the conclusion that PGItc could be suitable for cell culturing. Cell viability in PGItc + PLA extract medium was similar to that obtained from PGSu and PLLA nonwovens [61]. This was also confirmed in the study of PGCit-PLA nonwovens [64], demonstrating that PGItc + PLA nonwovens are likely to be significant in developing tissue engineering materials in the future. Further cytotoxicity studies of PGItc + PLA fibrils should be carried out, where the synthesized PGItc gel will have a higher degree of permeation or will be purified before the electrospinning process. As mentioned earlier, unreacted monomers may adversely affect cell survival.

The tested PGItc + PLA nonwovens showed similar thermal crosslinking efficiencies to other polyester nonwovens in which glycerol was one of the components—nonwovens made from poly(glycerol sebacate) and polylactide (a highly esterified product) [63]. A difference appeared in the crosslinking efficiency values obtained according to the ratio of the used glycerol polyester to polylactide [63]. In the case of PGItc + PLA nonwovens, the higher the proportion of PGItc, the lower the crosslinking efficiency. In contrast, the situation is reversed for PGS and PLLA nonwovens (products with a high degree of esterification) [63]. However, the correlation of the obtained PGItc + PLA nonwovens is the same as for PGS and PLLA nonwovens with lower conversion [63]. In earlier studies, PGItc was synthesized by a reaction between itaconic anhydride and glycerol [47]. The product synthesized by the reaction of substrates with a functionality of OH/COOH = 0.5 had the lowest degree of esterification [47]. This coincides with the higher weight loss values of crosslinked and uncrosslinked samples—more unreacted substrates are eluted.

For both PGItc + PLA nonwovens and PLA-cellulose nano-fibers, a peak originating from the cold crystallization temperature (T_cc2_) of PLA is present before the melting temperature T_m2_ is reached [70]. Frone A.N. et al. explained [70] the presence of this temperature by the increase in the presence of crystalline lamellae. These lamellae were formed as a result of the previously occurring cold crystallization [70]. In contrast to the nonwovens obtained from PLA-cellulose, the PGItc + PLA nonwovens showed a less prominent but visible peak originating from the glass transition temperature T_g2_ and the cold crystallization T_cc2_ during the second heating [70]. This implies that the first cooling did not provide a high degree of crystallinity in the nonwovens. The exceptions were the uncrosslinked nonwovens PGItc:PLA 25:75 (OH/COOH = 1.5), 50:50 (OH/COOH = 1) and 75:25 (OH/COOH = 0.5), and the crosslinked nonwovens PGItc:PLA 25:75 (OH/COOH = 0.5 and 1.5), which did not show the presence of T_cc2_. The difference between the presence and absence of T_g2_ and T_cc2_ peaks for the PGItc + PLA and PLA-cellulose nonwovens might be caused by the applied cooling rate [70]. In a paper where PLA-cellulose nonwovens were studied, the cooling rate was 2 °C/min and for the PGItc + PLA nonwovens 10 °C/min [71]. Like PLA-cellulose fibers, PGItc + PLA fibers show a difference in crystallization and melting enthalpy values [70]. This demonstrates the continuous changes in the structure of PLA small crystals [70,72].

Compared to PGSu-PLLA and PGSu-PLCL fibers, non-crosslinked PGItc + PLA fibers have higher glass transition temperatures [61]. In the case of PGItc + PLA fibers, this indicates a higher contribution of intermolecular interactions between the reactants used. The same pattern applies to cold crystallization temperatures [61]. The comparison of melting temperatures shows that PGItc + PLA nonwovens exhibit slightly lower T_m_ values than PGSu-PLLA fibers [61]. This might contribute to the slightly poorer mechanical properties of the material made from PGItc + PLA fibers than PGSu-PLLA. However, the T_m_ values of PGItc + PLA fibers are higher than the melting temperatures obtained for PGSu-PLLA fibers [61].

Compared to crosslinked PGS-PLLA fibers, most crosslinked PGItc + PLA fibers show the presence of cold crystallization transformation [63].

In the article [71], a thermal analysis of PLA and PBAT, poly(butylene adipate- co-terephthalate)-based nonwovens, was performed [71]. Similar to PGItc + PLA fibers, PLA-PBAT fibers showed a glass transition temperature of around 60 °C. There is a significant temperature difference between the PGItc + PLA and PLA-PBAT compositions when it comes to the cold crystallization temperature of PLA. In the case of PLA-PBAT nonwovens, cold crystallization of the PLA phase occurred between 105–115 °C [71]. In the case of non-crosslinked PGItc + PLA nonwovens, the cold crystallization process occurred twice—during the first (T_cc1_) and second (T_cc2_) heating of the nonwovens. The temperature T_cc1_ was close to the cold crystallization temperature of pure PLA [61]. Only during the second heating of the nonwovens did the cold crystallization temperature T_cc2_ reach values above 100 °C. This demonstrates the effect of the presence of PGItc on the mobility of PLA chains [71].

The melting enthalpy values obtained for the PGItc + PLA nonwovens at the lowest PGItc content are similar to those obtained for the PGSu-PLLA nonwovens [61]. As in the case of the PGSu-PLLA nonwovens, as the PGItc component content increases, the melting enthalpy values decrease.

## 4. Conclusions

In this work, nonwovens of poly(glycerol itaconate) and polylactide obtained by electrospinning were described for the first time. Their morphology, wettability, cytotoxicity and thermal properties were studied.

Three different PGItc:PLA ratios were investigated—25:75, 50:50 and 75:25. A higher PGItc gel content could negatively affect cell viability due to the high amount of unreacted monomers.

The most important conclusions are:

- The combination of poly(glycerol itaconate) and polylactide properties provides a non-cytotoxic material with cell viability above the cytotoxicity limit (>70%). PGItc can be used as fibers in subsequent investigations towards potential use in tissue engineering.
- The presence of unreacted monomers may have a negative impact on cell viability. In the future, conducting cytotoxicity studies of UV-crosslinked nonwovens will be beneficial to rearrange C=C bonds.
- The produced nonwovens were characterized as hydrophilic (contact angle < 90°). The higher proportion of poly(glycerol itaconate) and the lower OH/COOH ratio results in a product with better hydrophilic properties.
- Thermal crosslinking of PGItc + PLA nonwovens was successfully performed. Nonwoven leaching tests and DSC analysis of nonwoven samples confirmed it.
- The analysis of PGItc + PLA fibers made it possible to define the area of their potential application in tissue engineering. Based on the conducted studies, it was concluded that PGItc + PLA fibers could be used in the future as subcutaneous tissue fillers—for instance, for post-tumor defects, skin healing systems or as in vitro tumor models for cell-based compound screening (in drug discovery research).
- It appears to be interesting to test the potential use of PGItc in the form of hydrogels in medical applications.
- It is necessary to conduct further cytotoxicity studies (incubation time of 72 h) and mechanical tests.

## 5. Materials and Methods

### 5.1. PGItc Gels Synthesis Procedure

The syntheses were carried out in the Mettler Toledo MultiMax reactor system. The reactions were carried out in 50 mL Hastlelloy reactors. The substrates, glycerol (≥99%, Sigma Aldrich, Burlington, MA, USA) and itaconic anhydride (99%, Ambeed, Arlington Heights, IL, USA), were used without prior preparation. The reaction was performed under solvent-free conditions and without any catalyst. The substrates were weighed to the reactor in amounts depending on the molar ratio of the functional groups. The weight of the reagents used in the syntheses was 30 g. 

The reactors were provided with temperature sensors, mechanical stirrers and Dean–Stark instruments. In the first stage of the reaction, the mixture was heated for 20 min to a temperature of 140 °C. This temperature was kept constant for 5 h. After the reaction was finished, the mixture was cooled to room temperature. The parameters of the obtained products (the degree of esterification of ED_NMR_ and ED_tit_, and the degree of conversion of itaconic anhydride %X_13C_^NMR^) were described in a previous article about the synthesis of PGItc [49].

Table 2 summarizes the amounts of reactants used in the syntheses of PGItc that were performed.

### 5.2. FTIR Analysis

To perform IR analysis of the investigated samples, small amounts of the resulting polymer and standards were weighed into the vials using a technical balance. IR analyses were carried out using an ALPHA spectrometer from Bruker. The measurements used the technique of attenuated total reflection (ATR).

### 5.3. Nuclear Magnetic Resonance (NMR) Spectroscopy

In order to perform the NMR analysis of the test samples, 130.00 to 160.00 mg of the resulting polymer were weighed into vials on an analytical balance. Then, 1 mL of solvent—deuterated DMSO—was added. The prepared samples were firmly closed with a cap and placed on a Heidolph 545-10000-00 vibrating shaker to dissolve the vial’s contents. Then, a 700 μL sample was taken with an automatic pipette and loaded into a glass tube. After this, the prepared sample underwent NMR analysis. NMR spectra were performed using an Agilent 400 MHz spectrometer.

### 5.4. Electrospinning Procedure

Commercially available PLA (PA, Corbion Purasorb PL49—no longer available) was used in the solution with PGItc gels. The polymers’ ratios were 25:75, 50:50 and 75:25 (PGItc:PLA). Hexafluoroisopropanole (5% concentration) (HFIP, Iris Biotech) was used as a solvent. The prepared solutions were left to stir for 24 h. A rotary collector with a pre-set linear speed of 1 m/s was used for the electrospinning process. Electrospinning was held with 8.5–11.5 kV voltage. The solution dosing rate was set as 1.2 mL/h (chosen as a fixed value; electrospinning was possible in a wide range of dosing rates, from 0.5 mL/h to 3 mL/h). The distance between the needle and the collector was 14 cm. The low molecular weight of PGItc made it impossible to obtain nonwovens from PGItc only.

### 5.5. Scanning Electron Microscopy

A JEOL JSM-6010Plus/In-touch scope scanning electron microscope was used to characterize the morphology of the fibers obtained. To obtain SEM images, originally, non-conductive samples of polymeric materials were sputtered with a layer of gold. Afterwards, the samples were inscribed on the stand under vacuum conditions. Tests were performed at room temperature. The fiber diameter was estimated from the SEM images. To determine the fibers’ diameters, ImageJ software was used. The diameter value was averaged from ten randomly selected locations on the fiber surface.

### 5.6. Water Contact Angle Measurements

We used the sitting drop technique to investigate the wetting angle of the nonwoven surfaces. The surface of the solid forms the wetting angle under test and the tangent to the surface of the liquid droplet placed on it. In this study, it was deionized water. An OCA Physics optical goniometer was used for the measurements. The Industrial Digital Camera UCMOS01300KPA and Fixed Microscope Adapter FMA037 were used to measure the wetting angle. Toupview software was used to determine the wetting angle. The contact angle measurements were conducted in an air atmosphere. 

A drop of deionized water was applied to the surface of the fibers. After 5 s from the application of the drop, the wetting angle was measured. 

Five wetting angle measurements were obtained for each nonwoven. Three wetting angle measurements for each fiber were selected for further calculations. The wetting angle value for each nonwoven was an averaged value from the selected results. The error bars were determined as the standard deviation from the obtained measurement results.

To authenticate the results, wettability angles with iodomethane were measured for each nonwoven. The wetting angle for a drop of this substance was expected to be 0°. For each analyzed nonwoven, the wetting angle value was 0°.

### 5.7. Cytotoxicity Test

Cytotoxicity studies were performed using mouse fibroblast cell line L929 (ATCC).

DMEM culture medium with a glucose concentration of 1.0 g/L was used. The medium was supplemented with a 10% addition of fetal bovine serum and a 1% addition of antibiotics.

Sample preparation: discs (1.7 cm diameter) were cut from nonwoven fabrics and sterilized. Four discs were placed in each well; 1.3 mL of culture medium was added to each well, and the plate was incubated for 24 h at 37 °C.

After that, 100 μL of 10^5^ cells/mL cell suspension was placed in each well. The outer wells were flooded with DPBS buffer to prevent evaporation of the culture medium. For the following 24 h, the cells were incubated at 37 °C. After that, the culture medium was removed and replaced with the extracts from nonwovens (100 μL per well). To perform a negative control, some of the wells were flooded with fresh media only. The plate was incubated again for 24 h at 37 °C. 

After 24 h, all wells were washed twice with 100 μL of DPBS buffer. Afterwards, 150 μL of XTT reagent solution in DMEM was added to each well. The plate was incubated for another 4 h at 37 °C. After that, absorbance measurements (wavelength: 450 nm and 630 nm) were taken using the plate reader. The obtained results were analyzed. Outlier results were rejected based on the Q-Dixon test, and the remaining values were averaged.

The error bars were determined as the standard deviation from the obtained measurement results. 

### 5.8. Optical Profilometer Surface Imaging

Surface imaging was performed using a VEECO WYKO NT 9300 optical profilometer. The phenomenon of light interference was used. The non-contact mapping of the nonwoven surface contours was performed to avoid sample damage. 

A small section of the obtained non-woven fabric was placed on the measuring table, and then measurements were taken. Green and white LED light (λ = 67.4393 nm, 69.1379 nm, 72.4703 nm). Fifty measurements were taken for each sample. Lenses with 5×, 20× and 50× magnifications were used for the measurements. The Vertical Scanning Interferometry (VSI) mode of operation was used for the measurements. A 640 × 480 (pixel array) measurement matrix was used.

### 5.9. Crosslinking and Leaching of the Nonwovens

For each of the nonwovens, 10 small rectangular samples were prepared. Each nonwoven was weighed before further operations. 5 samples of each nonwoven underwent the crosslinking process. Crosslinking of nonwovens was carried out in a vacuum dryer. For the first 24 h, the samples were crosslinked at room temperature (approx. 23 °C) at 20 mbar. For the next 24 h, the temperature was raised to 40 °C, and the vacuum was applied again. For the last 24 h, the temperature was raised to 80 °C.

To determine the crosslinking efficiency of PGItc, 6 samples from each nonwoven (3 crosslinked and 3 uncrosslinked) were dissolved in 99.8% ethanol (with stirring, 48 h, 25 °C). The uncrosslinked PGItc was easily eluted from the sample. The proportion of crosslinked PGItc was determined by the percentage weight loss of the dried nonwoven samples (vacuum dryer, 48 h, 25 °C). The error bars were determined as the standard deviation from the obtained measurement results.

### 5.10. DSC Analysis

A Q2000 DSC analyzer (TA Instruments) was used to perform the DSC analysis. The analysis was performed on samples weighing approximately 5 mg. The applied sample mass was determined by the sensitivity of the analysis apparatus. The procedure for DSC analysis was as follows. The first step was to cool the sample to −50 °C. The specimen was then heated to 250 °C (10 °C/min step) (to characterize the electrospun material). In the next stage, the sample was again cooled to −50 °C. In the final fourth stage, the specimen was heated to 250 °C (to characterize the product of the reaction between PLA and PGItc). DSC thermograms were edited in TA Instruments Universal Analysis 2000 software.

## Figures and Tables

**Table 1:** Mean fiber diameters [μm].

PGItc:PLA Ratio	OH/COOH Ratio		
	0.5	1	1.5
25:75	3.08 ± 0.27	1.80 ± 0.08	3.28 ± 0.17
50:50	2.51 ± 0.24	2.00 ± 0.35	2.26 ± 0.05
75:25	1.74 ± 0.12	1.55 ± 0.24	1.26 ± 0.14

**Table 2:** Formulation of the reaction mixture.

Reactant	OH/COOH Ratio		
	0.5	1	1.5
Itaconic Anhydride (IAn)	23.55 g (0.210 mol)	19.38 g (0.173 mol)	16.47 g (0.147 mol)
Glycerol (G)	6.45 g (0.070 mol)	10.62 g (0.115 mol)	13.53 g (0.147 mol)

## Data Availability

The data presented in this study are available on request from the corresponding author.

## Supplementary Materials

The following supporting information can be downloaded at: https://www.mdpi.com/article/10.3390/gels9100788/s1, Figure S1: The form of the obtained PGItc product; Table S1: The weight of the non-crosslinked nonwovens before and after leaching; Table S2: The weight of the crosslinked nonwovens before and after leaching; Table S3: Characteristic temperatures and enthalpies for PGItc-PLA non-crosslinked and crosslinked nonwovens; Table S4: Degree of crystallinity of the samples after first heating [%]; Table S5: Degree of crystallinity of the samples after second heating [%].

## Abbreviations

ECM: extracellular matrix, FA: fluorapatite, Fac: fumaric acid, Gel: gelatin, GO: graphene oxide, PBAT: poly(butylene adipate-co-terephthalate), PCL: poly(ε-caprolactone), PCLF: polycaprolactone fumarate, PGCit: poly(glycerol citrate), PGDF: poly(glycerol-dodecanedioate-co-fumarate), PGItc: poly(glycerol itaconate), PGS: poly(glycerol sebacate), PGSu: poly(glycerol succinate), PLA: polylactide, PLCL: polycaprolactone lactide, PLLA: poly(L-lactide), PLGA: poly(lactide-co-glycolide) copolymer, PPF: poly(propylene fumarate), PPFcPM: poly(propylene fumarate-co-propylene maleate) copolymer, Si-Mg-FA: silicon and magnesium.
