# Supplementary material for: From Poly(glycerol itaconate) Gels to Novel Nonwoven Materials for Biomedical Applications
Source: Gels. 2023 Sep 29;9(10):788. doi: 10.3390/gels9100788 (PMC10606113; doi:10.3390/gels9100788)
Supplement: Supplementary file 1 [file gels-09-00788-s001.zip › gels-2616075-supplementary.pdf]

From Poly(glycerol itaconate) Gels to Novel Nonwoven Materials for Biomedical Applications

Magdalena Miętus [1], Krzysztof Kolankowski [1], Tomasz Gołofit [1], Piotr Denis [2], Aleksandra Bandzerewicz [1], Maciej Spychalski [3], Marcin Mąkosa-Szczygieł [4], Maciej Pilarek [5], Kamil Wierzchowski [5] and Agnieszka Gadomska-Gajadhur [1,*]

[1] Faculty of Chemistry, Warsaw University of Technology, Noakowskiego 3 Street, 00-664 Warsaw, Poland; magdalena.mietus.dokt@pw.edu.pl (M.M.); krzysztof.kolankowski.dokt@pw.edu.pl (K.K.); tomasz.golofit@pw.edu.pl (T.G.); aleksandra.bandzerewicz.dokt@pw.edu.pl (A.B.)

[2] Laboratory of Polymers and Biomaterials, Institute of Fundamental Technological Research, Polish Academy of Sciences, Pawińskiego 5B Street, 02-106 Warsaw, Poland; pdenis@ippt.pan.pl

[3] Faculty of Materials Science and Engineering, Warsaw University of Technology, Wołoska 141 Street, 02-507 Warsaw, Poland; maciej.spychalski@pw.edu.pl

[4] Faculty of Natural Sciences, Department of Chemistry, Norwegian University of Science and Technology, 7034 Trondheim, Norway; marcin.k.makosa-szczygiel@stud.ntnu.no

[5] Faculty of Chemical and Process Engineering, Warsaw University of Technology, Waryńskiego 1 Street, 00-645 Warsaw, Poland; maciej.pilarek@pw.edu.pl (M.P.); kamil.wierzchowski@pw.edu.pl (K.W.)

* Correspondence: agnieszka.gajadhur@pw.edu.pl

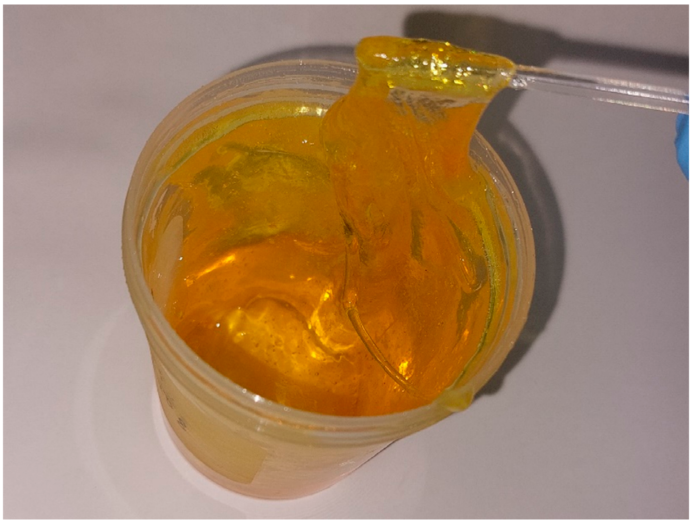

Figure S1. The consistency of the obtained PGItc product.

Table S1. The weight of the non-crosslinked nonwovens before and after leaching.

OH/COOH ratio	PGItc:PLA ratio	Weight before leaching [mg]	Weight after leaching [mg]	Loss of mass [mg]	%Loss of mass	Average %Loss of mass	Average %standard deviation of mass
0.5	75:25	2.37	0.71	1.66	70	70	1.5
		2.04	0.59	1.45	71		
		2.39	0.76	1.63	68		
	50:50	2.35	1.25	1.10	47	49	3.0
		2.27	1.11	1.16	51		
		2.12	1.02	1.10	52		
	25:75	3.99	2.90	1.09	27	27	0.5
		5.03	3.70	1.33	26		
		4.41	3.24	1.17	27		
1	75:25	8.31	2.32	5.99	72	71	1.0
		15.67	4.59	11.08	71		
		9.23	3.04	6.19	67		
	50:50	5.07	2.45	2.62	52	48	3.7
		4.08	2.15	1.93	47		
		7.98	4.45	3.53	44		
	25:75	1.63	1.13	0.50	31	31	0.8
		2.09	1.45	0.64	31		
		1.90	1.29	0.61	32		
1.5	75:25	5.03	3.70	1.33	26	69	1.4
		4.41	3.24	1.17	27		
		6.79	1.98	4.81	71		
	50:50	1.14	0.67	0.47	41	43	1.9
		3.10	1.77	1.33	43		
		1.31	0.72	0.59	45		
	25:75	2.55	1.73	0.82	32	33	2.1
		1.74	1.13	0.61	35		
		1.78	1.23	0.55	31		

Table S2. The weight of the crosslinked nonwovens before and after leaching.

OH/COOH ratio	PGItc:PLA ratio	Weight before leaching [mg]	Weight after leaching [mg]	Loss of mass [mg]	%Loss of mass	Average %Loss of mass	Average %standard deviation of mass
0.5	75:25	1.40	1.20	0.20	14.29	21.82	6.57
		3.99	3.00	0.99	24.81		
		4.02	2.96	1.06	26.37		
	50:50	1.27	1.13	0.14	11.02	13.85	3.69
		1.11	0.91	0.20	18.02		
		0.64	0.56	0.08	12.50		
	25:75	3.48	3.38	0.10	2.87	2.68	0.61
		2.83	2.74	0.09	3.18		
		1.00	0.98	0.02	2.00		
1	75:25	9.80	8.35	1.45	14.80	14.32	1.66
		12.17	10.26	1.91	15.69		
		11.70	10.24	1.46	12.48		
	50:50	1.20	1.12	0.08	6.67	8.48	4.05
		2.44	2.12	0.32	13.11		
		2.48	2.34	0.14	5.65		
	25:75	0.89	0.87	0.02	2.25	1.82	0.47
		1.52	1.50	0.02	1.32		
		1.05	1.03	0.02	1.90		
1.5	75:25	2.44	1.84	0.60	24.59	19.61	4.91
		2.30	1.96	0.34	14.78		
		3.03	2.44	0.59	19.47		
	50:50	3.04	2.47	0.57	18.75	19.21	0.44
		2.96	2.39	0.57	19.26		
		6.93	5.57	1.36	19.62		
	25:75	2.33	2.26	0.07	3.00	2.82	0.80
		1.99	1.92	0.07	3.52		
		2.06	2.02	0.04	1.94		

Table S3. Characteristic temperatures and enthalpies for PGItc-PLA non-crosslinked and crosslinked nonwovens.

Compared parameter	PGItc:PLA ratio	OH/COOH ratio					
		Non-crosslinked nonwoven			Crosslinked nonwoven		
		0,5	1	1,5	0,5	1	1,5
T_{g1} [°C]	25:75	2.8	-6.2	-23.2	-	-	-
	50:50	-2.3	-12.1	4.6	-	-	-
	75:25	2.1	-5.1	-10.3	-	-	-
$T_{g1'}$ [°C]	25:75	51.4	54.5	51.3	49.2	51.4	35.8
	50:50	58.1	58.3	55.2	53.0	52.1	42.2
	75:25	55.9	59.6	52.3	58.1	32.0	35.3
T_{g2} [°C]	25:75	61.9	62.4	-	61.0	61.9	61.3
	50:50	63.0	62.6	63.0	62.1	60.6	56.1
	75:25	-	63.5	61.8	-	61.1	53.4
T_{cc1} [°C]	25:75	71.0 (12.8 J/g)	71.5 (11.4 J/g)	70.0 (10.4 J/g)	-	-	-
	50:50	76.4 (22.8 J/g)	75.4 (15.2 J/g)	74.8 (13.5 J/g)	-	-	-
	75:25	76.6 (8.2 J/g)	79.1 (7.8 J/g)	75.4 (8.8 J/g)	-	-	-
T_{cc2} [°C]	25:75	107.6 (19.1 J/g)	104.5 (10.9 J/g)	-	103.5 (20.7 J/g)	106.4 (7.7 J/g)	101.0 (0.8 J/g)
	50:50	108.1 (7.1 J/g)	-	108.6 (7.3 J/g)	103.0 (2.9 J/g)	101.8 (5.4 J/g)	101.6 (6.0 J/g)
	75:25	-	112.1 (7.4 J/g)	99.3 (3.6 J/g)	105.2 (3.3 J/g)	103.3 (13.7 J/g)	104.5 (7.8 J/g)
T_{ccc} [°C]	25:75	99.5 (7.0 J/g)	102.3(5.3 J/g)	108.8 (29.1 J/g)	99.9 (5.8 J/g)	-	105.5 (30.2 J/g)
	50:50	103.1 (6.1 J/g)	131.1 (1.3 J/g) *105.1 (5.1 J/g)	101.9 (9.7 J/g)	102.7 (21.2 J/g)	104.1 (10.5 J/g)	106.6 (16.2 J/g)
	75:25	107.9 (15.4 J/g)	-	103.1 (7.6 J/g)	100.8 (5.4 J/g)	100.7 (8.5 J/g)	-
T_{c2} [°C]	25:75	162.2 (4.8 J/g)	161.5 (6.5 J/g)	-	158.5 (11.3 J/g)	161.0 (1.1 J/g)	-
	50:50	163.2 (2.1 J/g)	162.9 (1.9 J/g)	163.7 (5.6 J/g)	161.5 (1.7 J/g)	158.2 (2.0 J/g)	-
	75:25	-	165.2 (1.3 J/g)	162.8 (3.4 J/g)	163.6 (3.3 J/g)	156.1 (1.7 J/g)	-
T_{m1} [°C]	25:75	181.5 (40.0 J/g)	181.9 (38.4 J/g)	177.7 (39.7 J/g)	180.0 (50.0 J/g)	176.5 (10.2 J/g)	178.0 (40.6 J/g)
	50:50	176.1 (30.3 J/g) **180.6 (27.8 J/g)	177.5 (31.6 J/g) **181.6 (31.1 J/g)	178.3 (30.3 J/g)	176.8 (31.2 J/g)	179.2 (30.2 J/g)	179.3 (24.3 J/g)

	75:25	177.1 (14.9 J/g)	176.7 (12.9 J/g)	176.7 (16.9 J/g)	176.4 (18.4 J/g)	179.3 (38.1 J/g)	175.7 (11.4 J/g)
T_{m2} [°C]	25:75	179.2 (34.1 J/g)	177.7 (28.5 J/g)	175.9 (35.3 J/g)	174.0 (38.1 J/g)	172.7 (11.2 J/g)	167.9 (34.0 J/g)
	50:50	178.2 (25.6 J/g)	177.8 (24.6 J/g)	178.4 (25.7 J/g)	175.8 (27.3 J/g)	171.2 (22.6 J/g)	159.3 (22.4 J/g)
	75:25	178.6 (14.7 J/g)	177.7 (13.3 J/g)	175.9 (12.5 J/g)	176.4 (9.7 J/g)	169.4 (31.6 J/g)	153.3 (8.2 J/g)

The standard error of the determined enthalpy value for crosslinked samples was 2% from the enthalpy value obtained for uncrosslinked and crosslinked samples. The results marked in black are within the limits of the standard error. The results outside the limits of the standard error are marked in gray. In most cases, the values of the determined enthalpies for the tested samples are beyond the standard error which was assumed earlier. It means that the crosslinking of the samples affects the thermal properties of the tested nonwovens.

Where:
T_{g1} – First glass transition temperature during first heating (midpoint temperature);
$T_{g1'}$ – Second glass transition temperature during first heating (midpoint temperature);
T_{g2} - Glass transition temperature during second heating (midpoint temperature);
T_{cc1} – Cold crystallization temperature during first heating (peak temperature);
T_{cc2} – Cold crystallization temperature during second heating (peak temperature);
T_{c2} – Crystallization temperature during second heating (peak temperature);
T_{ccc} - Cold crystallization temperature during cooling (peak temperature);
T_{m1} - Melting temperature during first heating (peak temperature);
T_{m2} - Melting temperature during second heating (peak temperature);
*Second cold crystallization temperature during cooling (peak temperature);
*Second melting temperature during first heating (peak temperature).

Values for the enthalpy of cold crystallization (ΔH_{cc1}, ΔH_{cc2}), enthalpy of cold crystallization during cooling (ΔH_{ccc}) and enthalpy of melting (ΔH_{m1}, ΔH_{m2}) of investigated nonwovens are shown in brackets. The enthalpy values were determined by the onset method.

In order to determinate the degree of crystallinity of the samples, a following formula was used:

$$X_c = (\Delta H_{m1 \text{ or } 2} / \Delta H_m^{\circ}) \times 100\% \ [\%] \quad (1)$$

Where ΔH_m° is the specific enthalpy of melting of 100% crystalline PLA (93 J/g).

Table S4. Degree of crystallinity of the samples after first heating [%].

PGItc:PLA ratio	OH/COOH ratio					
	**Non-crosslinked nonwoven**			**Crosslinked nonwoven**		
	0.5	1	1.5	0.5	1	1.5
25:75	43.0	41.3	42.7	53.8	11.0	43.7
50:50	32.6	34.0	32.6	33.5	32.5	26.1
75:25	16.0	13.9	18.2	19.8	41.0	12.3

Table S5. Degree of crystallinity of the samples after second heating [%].

PGItc:PLA ratio	OH/COOH ratio					
	**Non-crosslinked nonwoven**			**Crosslinked nonwoven**		
	0.5	1	1.5	0.5	1	1.5
25:75	36.7	30.6	38.0	41.0	12.0	36.6
50:50	27.5	26.5	27.6	29.4	24.3	24.1
75:25	15.8	14.3	13.4	10.4	34.0	8.8
